# Insulin-Like Growth Factor Binding Proteins Increase Intracellular Calcium Levels in Two Different Cell Lines

**DOI:** 10.1371/journal.pone.0059323

**Published:** 2013-03-19

**Authors:** Danielle Seurin, Alain Lombet, Sylvie Babajko, François Godeau, Jean-Marc Ricort

**Affiliations:** 1 INSERM UMR_S938, Centre de Recherche Saint-Antoine, Paris, France; 2 INSERM U999, Le Plessis-Robinson, France; 3 INSERM U872, Laboratoire de Physiopathologie Orale Moléculaire, Paris, France; 4 LBPA, ENS de Cachan, CNRS, Cachan, France; 5 UMR204, Université Montpellier 2, Montpellier, France; Albert Einstein College of Medicine, United States of America

## Abstract

**Background:**

Insulin-like growth factor binding proteins (IGFBPs) are six related secreted proteins that share IGF-dependent and -independent functions. If the former functions begin to be well described, the latter are somewhat more difficult to investigate and to characterize. At the cellular level, IGFBPs were shown to modulate numerous processes including cell growth, differentiation and apoptosis. However, the molecular mechanisms implicated remain largely unknown. We previously demonstrated that IGFBP-3, but not IGFBP-1 or IGFBP-5, increase intracellular calcium concentration in MCF-7 cells (Ricort J-M et al. (2002) FEBS lett 527: 293–297).

**Methodology/Principal Findings:**

We perform a global analysis in which we studied, by two different approaches, the binding of each IGFBP isoform (i.e., IGFBP-1 to -6) to the surface of two different cellular models, MCF-7 breast adenocarcinoma cells and C2 myoblast proliferative cells, as well as the IGFBP-induced increase of intracellular calcium concentration. Using both confocal fluorescence microscopy and flow cytometry analysis, we showed that all IGFBPs bind to MCF-7 cell surface. By contrast, only four IGFBPs can bind to C2 cell surface since neither IGFBP-2 nor IGFBP-4 were detected. Among the six IGFBPs tested, only IGFBP-1 did not increased intracellular calcium concentration whatever the cellular model studied. By contrast, IGFBP-2, -3, -4 and -6, in MCF-7 cells, and IGFBP-3, -5 and -6, in C2 proliferative cells, induce a rapid and transient increase in intracellular free calcium concentration. Moreover, IGFBP-2 and -3 (in MCF-7 cells) and IGFBP-5 (in C2 cells) increase intracellular free calcium concentration by a pertussis toxin sensitive signaling pathway.

**Conclusions:**

Our results demonstrate that IGFBPs are able to bind to cell surface and increase intracellular calcium concentration. By characterizing the IGFBPs-induced cell responses and intracellular couplings, we highlight the cellular specificity and complexity of the IGF-independent actions of these IGF binding proteins.

## Introduction

Insulin-like growth factors, IGF-I and -II, regulate many cellular processes such as proliferation, differentiation and survival through their association and activation of the type I IGF receptor (IGF-IR) (reviews in [1], [2]). In all biological fluids, IGFs are specifically associated with one of their six binding proteins, IGFBPs [2], [3]. Due to their high affinity towards IGFs, IGFBPs act not only as carriers that prolong IGFs’ half-lives, but also regulate the bioavailability of the growth factors to their cellular targets. In recent years, many pieces of evidence indicate that, unrelated to their ability to bind IGFs, these binding proteins also possess intrinsic intracellular properties such as the capacity to regulate cell growth, differentiation and apoptosis (for review [4]). IGFBPs appear to exert their IGF-independent biological actions through their interaction with a variety of binding partners localized at the surface or even within the cells (for review [5]). Nevertheless, the physiological significance of many of these interactions remains to be established as well as the signaling pathways regulated downstream of these binding targets. However, because IGFBPs expression is altered in several pathologies such as tumors and metabolic disorders [6]–[8], knowledge of the precise signaling pathways modulated by these proteins is still therefore of primary importance.

Calcium is a crucial element in cell physiology and a fine regulation of its concentration is absolutely necessary for cell viability. Thus, an increase in free cytosolic calcium may modulate the activity of a wide variety of calcium-binding proteins which are implicated in a considerable number of cellular effects (for review [9], [10]). In addition, changes in intracellular calcium concentrations represent an important cue in the induction of apoptosis, a cell process shown as being regulated by some IGFBPs (for review [11]). By inducing calcium release from endoplasmic reticulum compartment through phospholipase C activation, heterotrimeric G proteins coupled to transmembraneous receptors provide a link between extracellular signals and the intracellular domain. These proteins form a large family whose some members possess a specific sensitivity with respect to bacterial toxins such as pertussis or cholera toxins. In this regard, we previously showed that IGFBP-3, but neither IGFBP-5 nor IGFBP-1, transiently increased intracellular calcium concentration in an adenocarcinoma-derived breast cancer cell line, MCF-7 cells, by a pertussis-toxin sensitive signaling pathway [12].

In search of the molecular mechanisms regulated by IGFBPs, we therefore developed a systematic prospective study in order to extend our previous results [12] to other family members as well as to another cell model, providing a wide prospective study about the IGF-independent effects of IGFBPs in two different cell lines.

## Materials and Methods

### Antibodies and Materials

IGFBP-1 was from Calbiochem (La Jolla, CA), N(109)D-rhIGFBP-3 was from Upstate Biotechnologies (Lake Placid, NY), IGFBP-4 was a generous gift of Dr Zapf (Zurich, Switzerland). Antibodies to IGFBP-1, -2, -4 and -6 were from Santa Cruz Biotechnology (Santa Cruz, CA), antibodies to IGFBP-3 (*E. coli*) were a specific rabbit antiserum raised in our laboratory, and antibodies to IGFBP-5 were from Millipore (Molsheim, France). Alexa Fluor 488 goat anti-rabbit IgG antibodies and Alexa Fluor 633 rabbit anti-goat IgG antibodies were from Molecular Probes (Eugene, OR). All other biochemicals were from Sigma (Saint-Quentin Fallavier, France) or ICN (Orsay, France).

### Cell Culture

The MCF-7 breast adenocarcinoma-derived cell line (from American Type Culture Collection) and the previously described C2 myoblast cell line [13] were grown to 85–90% confluence in Dulbecco’s modified Eagle’s medium supplemented with 10% fetal calf serum and 100 units/ml penicillin and 100 µg/ml streptomycin. Before each experiment, cells were starved in media without serum for 16–24 hours.

### Construction of the IGFBP-2, -5 and -6-expression Vectors

The hIGFBPs cDNA encoding the mature proteins lacking signal peptide were amplified by polymerase chain reaction (PCR) using the 5′CGCGGATCCGAGGTGCTGTTCC3′ (forward) and 5′CGCGAATTCGGCTGCGGTCTACTG3′ (reverse), the 5′CGCGGATCCCTGGGCTCCTTCGTGCAC3′ (forward) and 5′CGCGAATTCGGACGCATCACTCAACGTTG3′ (reverse), and the 5′CGCGGATCCCGGTGCCAGGC3′ (forward) and 5′CGCGAATTCGGTTTGACCCCAAGC3′ (reverse) oligonucleotides for IGFBP-2, -5 and -6, respectively. These were inserted into the pGEX-6P-1 expression vector from Amersham Pharmacia Biotech, following digestion with BamH1 and EcoR1. The orientation of the inserts was confirmed by restriction mapping and sequencing was carried out to confirm that no errors had been introduced during the PCR steps.

### Preparation and Purification of IGFBPs

rIGFBP-2, -5, -6 or glutathione-S-transferase (GST) were purified as previously described [14]. Briefly, 500 mL cultures of heat-shock transformed Escherichia coli BL21 bacteria were induced by overnight treatment with 0.1 mM isopropyl-beta-D-thiogalactopyranoside (IPTG) at an optical density (OD) = 1 to 600 nm. The pellet was resuspended in 10 mL of 10 mM Tris-HCl pH 8, 150 mM NaCl, 1 mM EDTA containing a cocktail of protease inhibitors (Boehringer, Mannheim, Germany), then incubated for 15 min at 4°C in 100 µg/mL lysozyme, 5 mM DTT, 1.5% sarkosyl. Thereafter, the bacteria were lysed by sonication until the medium became translucid. The supernatant recovered after centrifugation was incubated overnight at 4°C with 5% glutathione Sepharose. Thereafter, the glutathione Sepharose beads were rinsed twice in PBS and either incubated with 50 mM Tris-HCl pH 8, 10 mM glutathione (Boehringer) to elute the GST, or resuspended in 1 mL 50 mM Tris-HCl pH 7, 150 mM NaCl, 1 mM EDTA, 1 mM DTT for separation of IGFBPs by incubation with 20 units of Precision Protease (Amersham Pharmacia Biotech) for 4h at 4°C. Proteins were quantified by the Bradford method using the Bio-Rad Protein Assay (BioRad, Hercules, CA, USA).

### Determination of Intracellular free Ca^2+^ Concentration

Changes in intracellular Ca^2+^ concentration in response to IGFBPs were measured using a QuantiCell 700 dynamic imaging microscopy system (Visitech Int. Ltd., UK), with 30–40 MCF-7 breast carcinoma cells or C2 myoblastic cells per field as previously described [15]. The cells were cultured on glass coverslips, washed, and incubated at 37°C for 60 min in PBS-calcium-free HEPES medium (20 mM HEPES/Tris, pH 7.4, 5.4 mM KCl, 2 mM Na_2_PO_4_, 0.8 mM MgCl_2_, and 5 mM glucose) containing 5 µM Fura-2/AM. Before analysis, the cells were washed twice with the same buffer. After background recording for 40 seconds (20 images), the experiment was initiated by adding different concentrations (2–50 nM) of IGFBP-1 to -6. Fluorescence images were obtained at intervals of 2 seconds and intracellular Ca^2+^ concentrations were calculated over 200 or 300 seconds by illuminating the culture by a xenon lamp equipped with a rotating wheel permitting alternate excitation at 340 and 380 nm. The ratio (***R***) of the 340–380 fluorescences (F_340_/F_380_) (filter centered at 510 nm) was continuously recorded. The equation [Ca] = (R-R_min_)/(R_max_-R)Sf*Kd can be used to convert the Fura-2 ratio values to intracellular calcium concentrations (where [Ca] is the calcium concentration, R is the Fura-2 340/380 ratio, R_min_ and R_max_ are the 340/380 ratios in the absence of calcium or in the presence of a saturating concentration of calcium respectively, and Sf*Kd is the product of the Kd of Fura-2 and a scaling value). The Fura-2 ratio values were measured as a function of calcium concentrations by using a calibration kit (Invitrogen - Molecular Probes). The dose response curves were obtained as follows. Cells cultured onto coverslips were incubated with different IGFBP concentrations (2, 5, 20 or 50 nM) and intracellular calcium concentration measured as previously described. Then, intracellular calcium concentration was obtained by integrating the area under the curve (measuring Ca^2+^ transient peaks plotted as a function of time for each field from the addition of one IGFBP until the end of image recording, 200 s) and averaging the fluorescence from the whole field of cells chosen.

### Confocal Fluorescence Microscopy

MCF-7 cells cultured onto coverslips were incubated for 30 min with HBS pH 7.4 containing 3% BSA and, then, incubated with or without IGFBP-1 to -6 (5 µg.mL^-1^) for 1 hour at 4°C. Cells were fixed in 3.7% (w/v) paraformaldehyde in PBS (BD Biosciences), then washed and incubated for 1 hour at 37°C with 5–10 µg.mL^-1^ of the desired MAbs and then for 30 min at 37°C with 1 µg of Alexa-488 conjugated goat anti-rabbit immunoglobulin G (IgG) or Alexa-633 conjugated rabbit anti-goat IgG heavy plus light chains (H+L) (Molecular Probes, Eugene, Oreg.). Cells were rinsed six times with HBS-BSA 3% and mounted with Mowiol antifading reagent (Calbiochem). Confocal microscope analysis was carried out using the TCS NT confocal imaging system (Leica Instruments, Heidelberg, Germany), equipped with a 63× objective (plan apo, numerical aperture = 1.4). For Alexa-488 or Alexa-633, an argon-krypton or helium-neon ion laser adjusted to 488, or 633 nm, respectively, was used. The signal was integrated over four to eight frames to reduce the noise. The pinhole was adjusted to allow a field depth of about 1 µm, corresponding to the increment between two adjacent sections. At least 20 cells were observed in each condition.

### Flow Cytometry

Cell surface binding of IGFBPs was assayed by FACS. Overnight serum starved cells were washed twice with ice cold PBS and harvested in ice cold PBS containing 2.5 mM EDTA. Cells were gently dissociated (by pipetting up and down the cell suspension), washed twice in ice cold PBS and incubated for 30 min at 4°C in PBS containing 5% BSA. Cells were incubated for 1 hour at 4°C with or without IGFBP-1 to -6 (5 µg.mL^-1^), then washed in PBS before fixation for 10 min at room temperature with 3.7% (w/v) paraformaldehyde in PBS. Non-specific binding sites were saturated by incubating cells in PBS containing 5% BSA and 1% goat serum. Cells were incubated for 1 h at room temperature with specific primary antibodies directed against IGFBP-1 to -6 diluted in last buffer, then washed three times before being incubated for 45 min at room temperature with secondary antibodies diluted in last buffer. After two washes, cell fluorescence was analyzed by flow cytometry with a FACSCalibur (BD Biosciences), and mean fluorescence intensities (MFI) were quantified using the CellQuest Pro software (BD Biosciences).

## Results

### IGFBP-2, -4 and -6 Specifically Modulate Intracellular Calcium Concentration in MCF-7 and C2 Proliferative Cells

**Figure 1:**
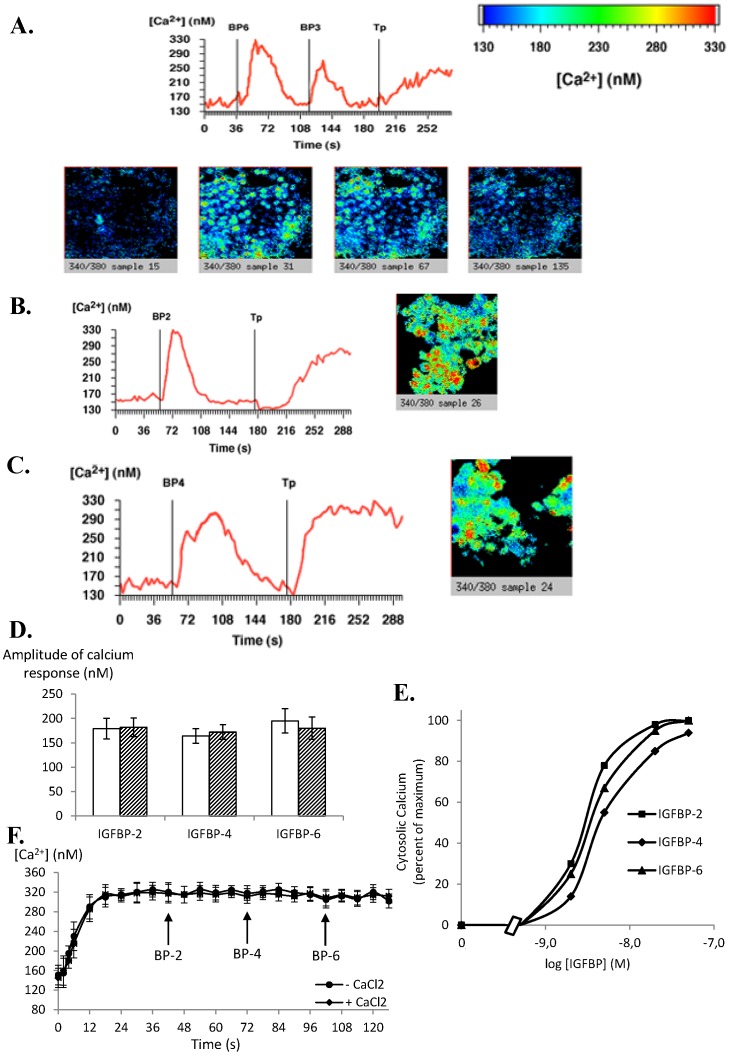
IGFBP-2, -3, -4 and -6 increase intracellular calcium concentrations in MCF-7 cells. MCF-7 cells cultured on glass coverslips were incubated with Fura-2/AM. A. After background recording for 40 seconds to determine basal intracellular calcium concentrations as described in Methods, cells were incubated with IGFBP-6 (20 nM), then with IGFBP-3 (20 nM) and then with thapsigargin (Tp) (1 µM). The results of a typical experiment are shown in which the whole field (red line) was analysed and intracellular calcium quantified. The slides from left to right show representative views of the cells (a) before addition of IGFBP-6, (b) after addition of IGFBP-6, (c) after addition of IGFBP-3, and (d) after addition of thapsigargin. The results presented are representative of 5 independent experiments. B and C. Same experiments as in panel A excepted that cells were incubated with either 20 nM IGFBP-2 (panel B) or 20 nM IGFBP-4 (panel C). Slides show representative views of the cells after addition of IGFBP-2 or -4, respectively. Results presented are representative of 4 independent experiments. D. Quantitative analysis of the calcium response (maximal calcium response - basal calcium level) obtained for IGFBP-2, -4 and -6 in calcium free (empty bars) or calcium containing (hatched bars) medium. Results are the means ± SEM for three to five independent experiments. E. Dose-response curves for intracellular calcium concentrations were established as described in Materials and Methods. Values are expressed as percentages of the maximal response measured with 50 nM IGFBP-2 and are the mean for two independent experiments. F. Graphs present the average values of the intracellular calcium concentration modulated by addition of 20 nM of IGFBP-2, -4 or -6 following thapsigargin (1 µM) treatment in calcium free or containing medium as indicated.

We previously demonstrated that IGFBP-3, but not IGFBP-1 or IGFBP-5, specifically regulated intracellular calcium concentration in MCF-7 cells [12]. We therefore wanted to determine whether the three other IGFBPs, i.e. IGFBP-2, -4 and -6, can also regulate cytosolic calcium concentration in these cells. Addition to MCF-7 cells of 20 nM (concentration chosen as a common maximal inducing response (Figure 1E) and in accordance with circulating or extracellular physiological concentrations) of IGFBP-6 (Figure 1A), IGFBP-2 (Figure 1B) and IGFBP-4 (Figure 1C) increased intracellular free calcium concentration. These responses were both rapid (occurring within 2–4 s) and transient. In fact, a return to basal levels occurred 50–70 s later for IGFBP-6 (Figure 1A) and IGFBP-4 (Figure 1C) and 30 s later for IGFBP-2 (Figure 1B). Quantitative analysis of the amplitude of the IGFBPs-induced calcium response in MCF-7 cells shows no significant difference between IGFBP isoforms and whatever the presence or the absence of extracellular calcium (Figure 1D). As shown in Figure 1E, the responses were dose-dependent: a significant response was yet observed with 1–2 nM IGFBP and was maximal using 20–50 nM IGFBP. Moreover, IGFBPs addition following thapsigargin treatment did not further increase intracellular calcium concentration whatever the incubation medium (calcium free or not) used (Figure 1F). The response of MCF-7 cells to 20 nM IGFBP-3 was confirmed (Figure 1A) and was similar as previously described [12]. Moreover, whatever the IGFBP combination used (i.e. first IGFBP-x and then IGFBP-y, or first IGFBP-y and then IGFBP-x), no difference in terms of the amplitude or absence of calcium responses was induced by IGFBP.

**Figure 2:**
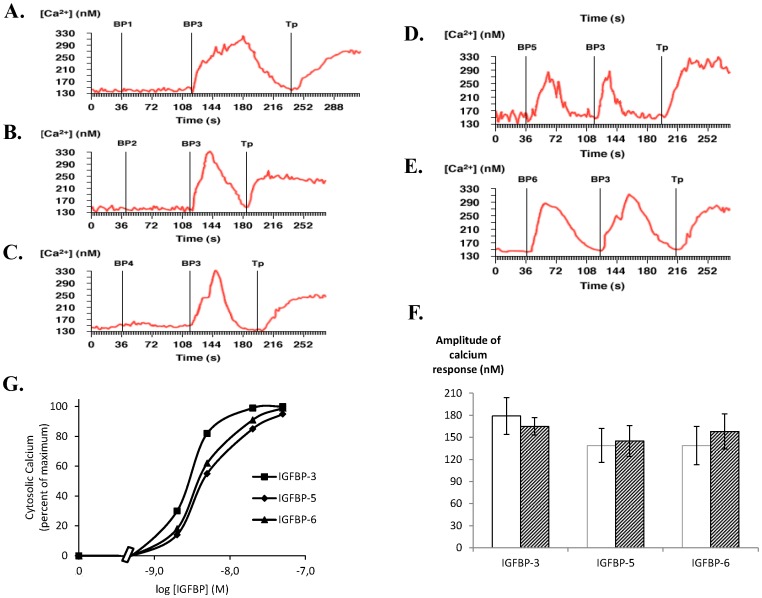
IGFBP-3, -5 and -6, but not IGFBP-1, -2 and -4, increase intracellular calcium concentration in C2 proliferative cells. **A-E.** C2 cells cultured on glass coverslips were incubated with Fura-2/AM. After background recording for 40 seconds to determine basal intracellular calcium concentrations as described in Methods, cells were incubated with either 20 nM IGFBP-1 (panel A), IGFBP-2 (panel B), IGFBP-4 (panel C), IGFBP-5 (panel D) or IGFBP-6 (panel E), then with IGFBP-3 (20 nM) and then with thapsigargin (1 µM). The results of a typical experiment are shown in which the whole field (red line) was analysed and intracellular calcium quantified. Results presented are representative of 3 independent experiments. F. Quantitative analysis of the calcium response (maximal calcium response - basal calcium level) obtained for IGFBP-3, -5 and -6 in calcium free (empty bars) or calcium containing (hatched bars) medium. Results are the means ± SEM for three independent experiments. G. Dose-response curves for intracellular calcium concentrations were established as described in Materials and Methods. Values are expressed as percentages of the maximal response measured with 50 nM IGFBP-3 and are the mean for two independent experiments.

Since the determination of cell specificity is of crucial importance for the study of cellular responses, we asked whether IGFBPs may similarly affect intracellular calcium concentrations in another cell model, C2 proliferative myoblast cells. Such cellular model was chosen due to the significant role of calcium in the myogenic lineage. In C2 cells, addition of 20 nM of IGFBP-3 (Figure 2, panels A-F), IGFBP-5 (Figure 2D) and IGFBP-6 (Figure 2E) increased intracellular free calcium concentration with similar amplitude and whatever the presence or absence of calcium in the extracellular medium (Figure 2F). As observed in MCF-7 cells, these responses were both dose-dependent (Figure 2G), rapid (occurrence 2–4 s) and transient with returns to basal values similar for each IGFBP-inducing response (30–40 s after maximal response). Whatever the concentration used (up to 200 nM) neither IGFBP-1 nor -2 nor -4 affected intracellular calcium concentration (Figure 2, panels A-C) demonstrating that cell response was specific to only three IGFBP isoforms.

Since both cell lines were incubated in calcium free medium and since no significant difference in terms of calcium increase was observed when the assays were performed in calcium containing medium (Figure 2F), we can conclude that calcium was released from intracellular stores into the cytoplasm and then re-absorbed. The viability of the cells in terms of calcium response was confirmed by their reaction to thapsigargin (1 µM) which induced total and irreversible calcium release from intracellular stores (Figures 1 and 2). As demonstrated in MCF-7 cells (Figure 1F), addition of either IGFBP-3, -5 or -6 after thapsigargin treatment did not further increase intracellular calcium concentration (data not shown). Moreover, addition to the cells of equal volumes of buffers that served to dilute or resuspend IGFBPs, never affected intracellular calcium concentration (data not shown) demonstrating that the cellular responses observed were specifically induced by IGFBPs.

### Intracellular Calcium Concentration Increase Induced by IGFBP-2 in MCF-7 Cells, and by IGFBP-5 in C2 Cells, Implies a Pertussis Toxin Sensitive Signaling Pathway

Rapid and transient increase in intracellular calcium concentration is commonly the consequence of heterotrimeric G protein activation. Therefore, we checked whether a G protein inhibitor, i.e. pertussis toxin, may block such IGFBP-induced cytosolic calcium increase.

**Figure 3:**
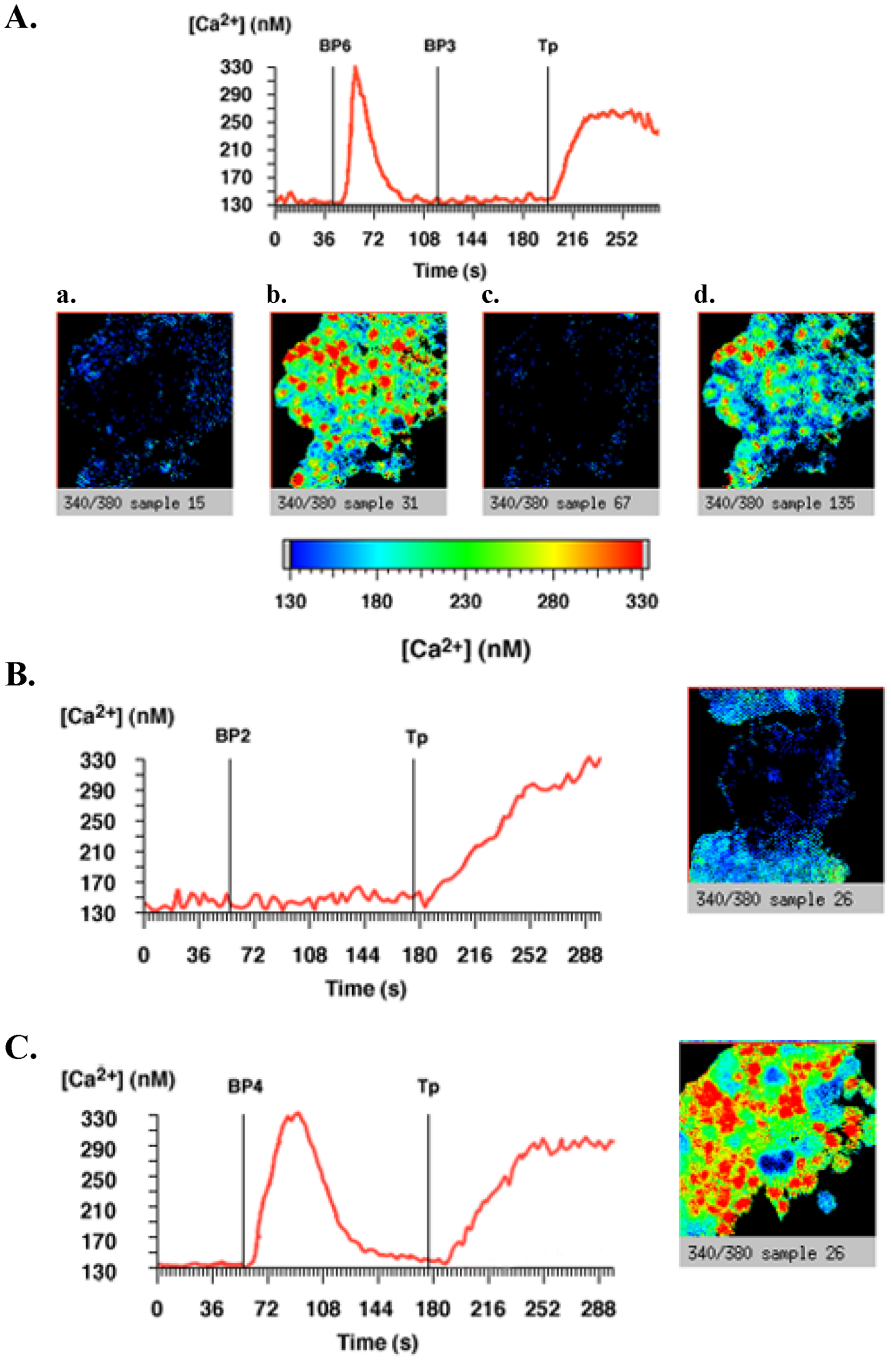
IGFBP-2 and -3, but not IGFBP-4 and -6, increase intracellular calcium concentrations by a pertussis toxin-sensitive signaling pathway in MCF-7 cells. MCF-7 cells cultured on glass coverslips were treated with 200 ng.mL^-1^ pertussis toxin (PTX) for 16 h at 37°C and were incubated with Fura-2/AM. A. After background recording for 40 seconds to determine basal intracellular calcium concentrations as described in Methods, cells were incubated with 20 nM IGFBP-6, then with IGFBP-3 (20 nM) and then with thapsigargin (Tp) (1 µM). The results of a typical experiment are shown in which the whole field (red line) was analysed and intracellular calcium quantified. The slides from left to right show representative views of the cells (a) before addition of IGFBP-6, (b) after addition of IGFBP-6, (c) after addition of IGFBP-3, and (d) after addition of thapsigargin. The results presented are representative of 4 independent experiments. B and C. Same experiments as in panel A excepted that cells were incubated with either 20 nM IGFBP-2 (panel B) or 20 nM IGFBP-4 (panel C). The results of a typical experiment are shown in which the whole field (red line) was analysed and intracellular calcium quantified. Slides show representative views of the cells after addition of IGFBP-2 or -4, respectively. Results presented are representative of 4 independent experiments.

**Figure 4:**
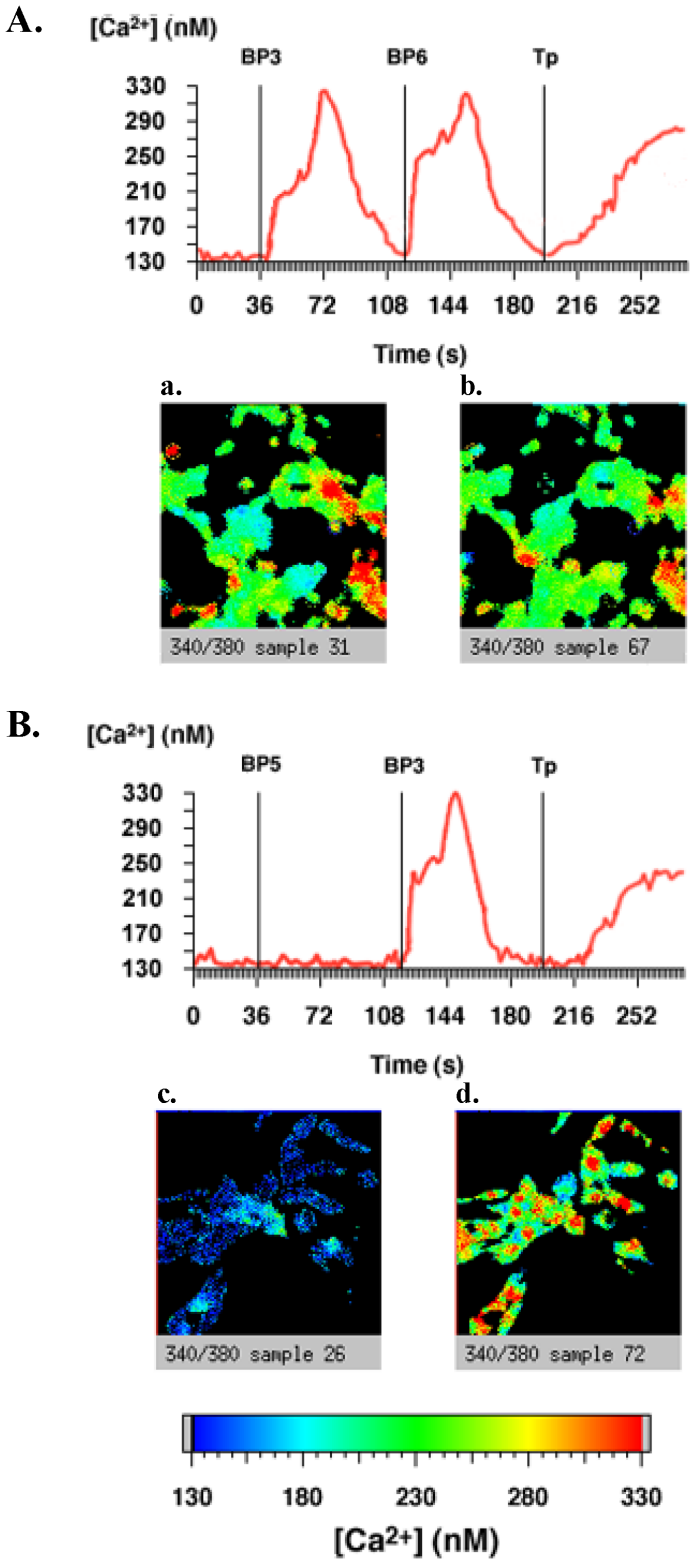
IGFBP-5, but not IGFBP-3 and -6, increases intracellular calcium concentrations via a pertussis toxin-sensitive signaling pathway in C2 cells. C2 cells cultured on glass coverslips were treated with or without 200 ng.mL^-1^ pertussis toxin (PTX) for 16 hours at 37°C and incubated with Fura-2/AM. After background recording for 40 seconds to determine basal intracellular calcium concentrations as described in Methods, cells were incubated (A) with IGFBP-3 (20 nM), then IGFBP-6 (20 nM) and then with thapsigargin (1 µM) or incubated (B) with IGFBP-5 (20 nM), then IGFBP-3 (20 nM) and then with thapsigargin (1 µM). The results of a typical experiment are shown in which the whole field (red line) was analysed and intracellular calcium quantified. The slides from left to right show representative views of the cells: panel A (a) after addition of IGFBP-3, (b) after addition of IGFBP-6, panel B: (c) after addition of IGFBP-5, and (d) after addition of IGFBP-3. Results presented are representative of 4 independent experiments.

In MCF-7 cells, pertussis toxin totally inhibited IGFBP-2-increased calcium concentration (Figure 3B) but did not impair IGFBP-4 (Figure 3C) and IGFBP-6 (Figure 3A) responses. As previously shown [12], pertussis toxin also blocked IGFBP-3-induced intracellular calcium concentration increase in MCF-7 cells (Figure 3A). In C2 proliferative cells, pertussis toxin totally inhibited IGFBP-5-increased cytosolic calcium (Figure 4B) but was without any effect on IGFBP-3 and -6 responses (Figure 4, panels A and B).

Taken together, these results showed that IGFBP-5 in C2 cells, and IGFBP-2 and -3 in MCF-7 cells, increase intracellular calcium concentration by a pertussis-toxin-sensitive intracellular signaling pathway. Moreover, they clearly pointed out that, depending on a given cell type, IGFBPs use specific and distinct intracellular signaling pathways to regulate cytosolic calcium concentration.

### IGFBPs Bind to the Plasma Membrane of MCF-7 Cells

**Figure 5:**
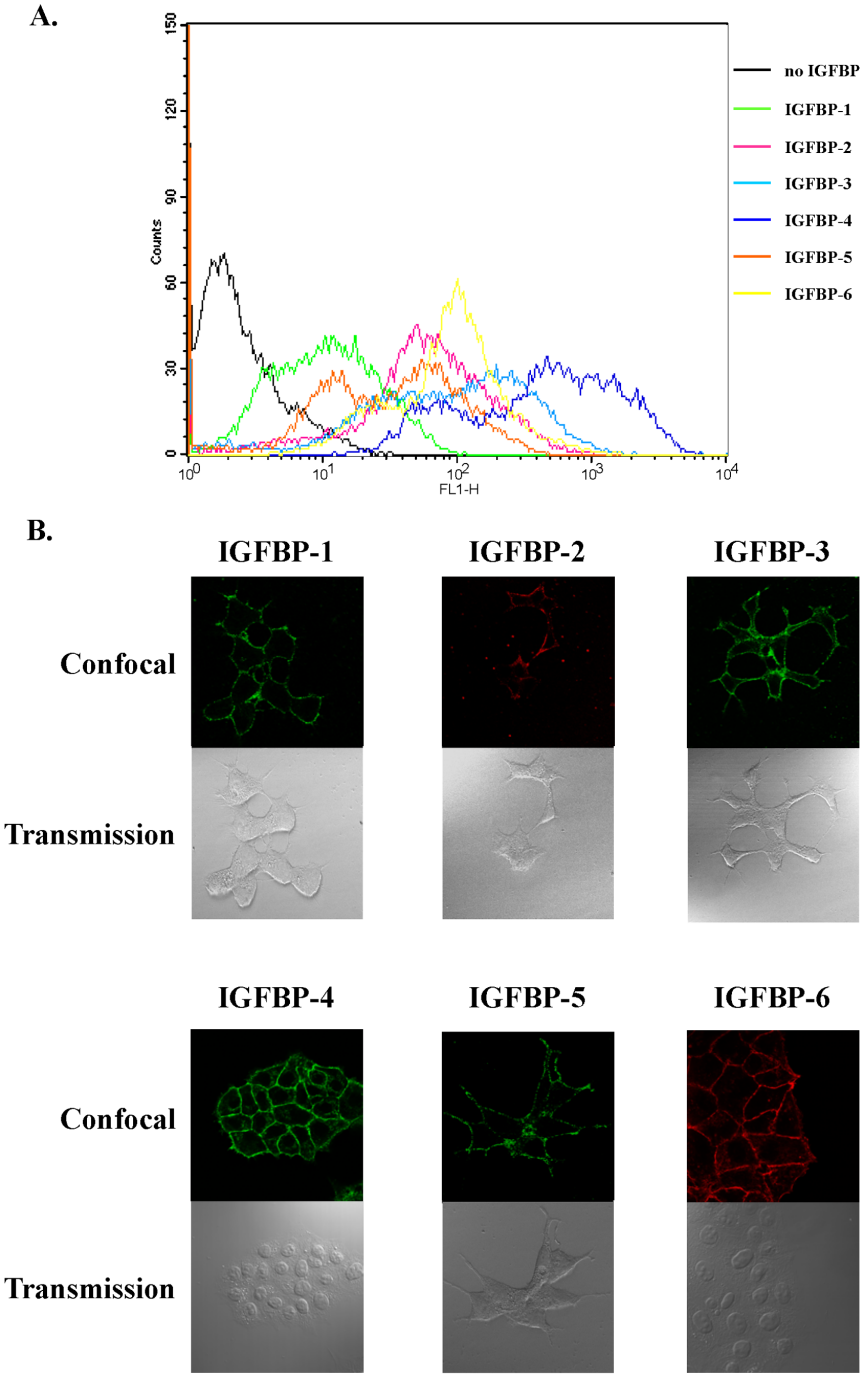
IGFBP-1 to -6 associate with MCF-7 cell surface. A. MCF-7 cells were incubated for 1 hour at 4°C with or without IGFBP-1 to -6 (5 µg.mL^-1^), fixed and incubated with specific primary antibodies and fluorescent secondary antibodies as indicated in Materials and Methods. Staining profiles were analyzed by FACS and mean fluorescence intensities (MFI) quantified. Data presented are representative of four independent experiments. B. MCF-7 cells growing on coverslips were incubated with IGFBP-1 to -6 (5 µg.mL^-1^) and then fixed without detergent. Cells were incubated with specific antibodies directed against IGFBP-1 to -6 processed with secondary antibodies stained either with Alexa 488 (IGFBP-1, -3, -4, -5) or Alexa 633 (IGFBP-2, -6). For each incubation condition, upper panel shows confocal fluorescent images and lower panel shows differential interference contrast images. Results are representative of three independent experiments.

**Figure 6:**
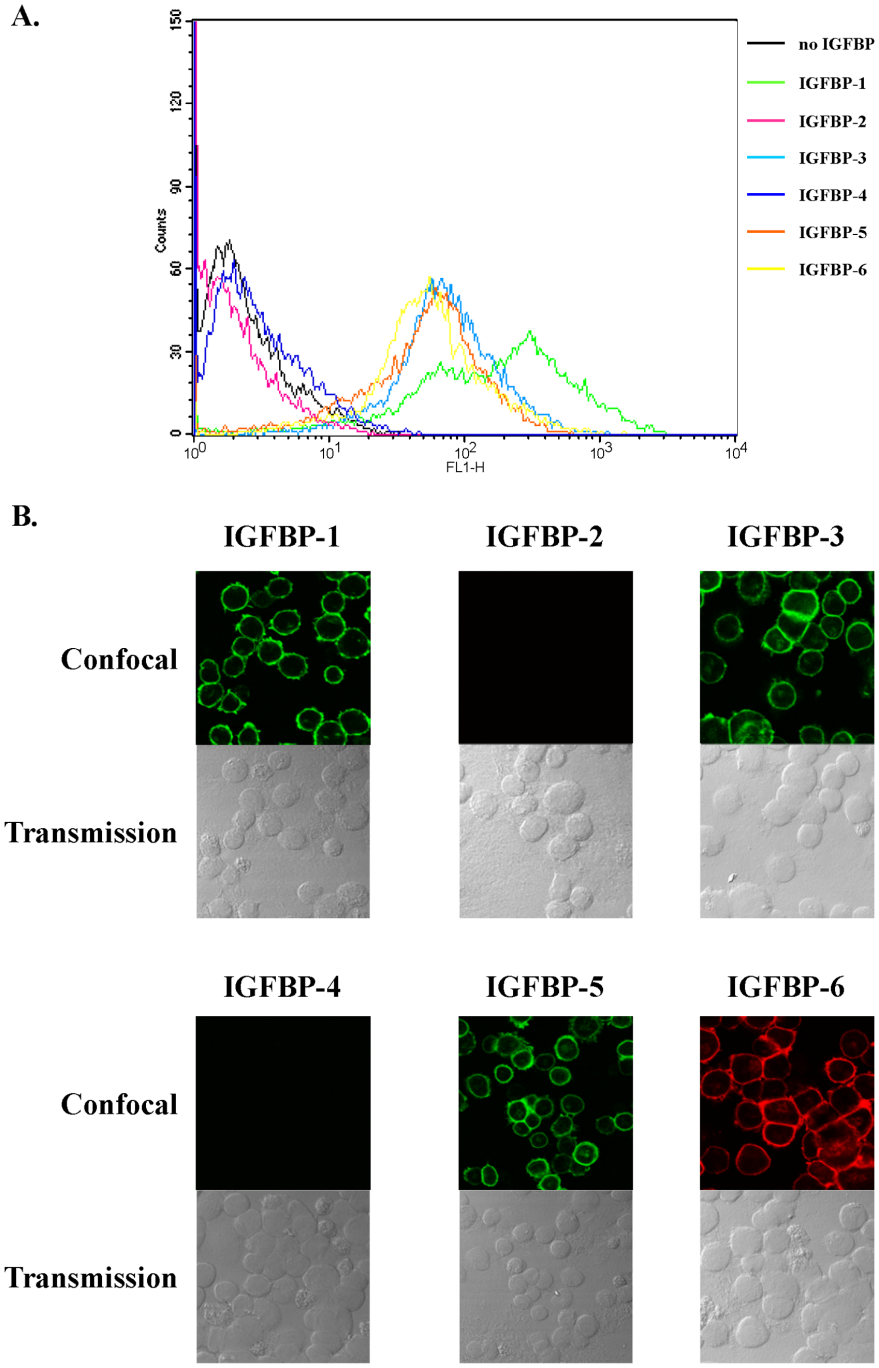
IGFBP-1, -3, -5 and -6, but not IGFBP-2 and -4, associate with C2 cell surface. A. C2 cells were incubated for 1 hour at 4°C with or without IGFBP-1 to -6 (5 µg.mL^-1^), fixed and incubated with specific primary antibodies and fluorescent secondary antibodies as indicated in Materials and Methods. Staining profiles were analyzed by FACS and mean fluorescence intensities (MFI) quantified. Data presented are representative of three independent experiments. B. C2 cells growing on coverslips were incubated with IGFBP-1 to -6 (5 µg.mL^-1^) and then fixed without detergent. Cells were incubated with specific antibodies directed against IGFBP-1 to -6 processed with secondary antibodies stained either with Alexa 488 (IGFBP-1, -3, -4, -5) or Alexa 633 (IGFBP-2, -6). For each incubation condition, upper panel shows confocal fluorescent images and lower panel shows differential interference contrast images. Results are representative of four independent experiments.

Since previous cellular responses were rapid, internalization processes would be improbable and we hypothesized that, as previously shown for IGFBP-3 in MCF-7 cells [12], IGFBPs that induced calcium response would bind to the cell surface. Therefore, by using both confocal microscopy and fluorescent-activated cell sorting (FACS), we set out to characterize the binding of IGFBP-2, -4 and -6 and that of IGFBP-3, -5 and -6 to the surface of MCF-7 and C2 cells, respectively. FACS analysis demonstrated that IGFBP-2, -4 and -6 bound to non permeabilized MCF-7 (Figure 5A) and that IGFBP-3, -5 and -6 associated to C2 cell surface (Figure 6A). Confocal analysis confirmed and strengthened these observations showing a clear plasma membrane detection of IGFBP-2, -4 and -6 on MCF-7 cells (Figure 5B) and of IGFBP-3, -5 and -6 on C2 cells (Figure 6B).

Although some IGFBPs do not regulate intracellular calcium concentration in MCF-7 and in C2 cells, we asked whether these isoforms could however bind to the cell surface. IGFBP-1 and -5 associated to the surface of non permeabilized MCF-7 cells (Figure 5A) and were visualized at the level of the plasma membrane (Figure 5B). Similar results were obtained for IGFBP-1 in C2 cells (Figure 6, panels A and B). By contrast, association and binding of IGFBP-2 and -4 were never detected on C2 cell surface (Figure 6, panels A and B). According to our previous data [12] and as expected, IGFBP-3 was shown to bind with MCF-7 cell surface (Figure 5, panels A and B).

## Discussion

In this study, we performed for the first time a wide prospective analysis of intracellular calcium responses induced by IGFBPs in two different cell models. Such responses were always rapid and transient, and occurred in a calcium-free medium indicating that calcium release comes from intracellular stores (i.e. endoplasmic reticulum) and dismissing the hypothesis of an IGFBP internalization-dependent mechanism. Thus, although some IGFBPs such as IGFBP-2 (for review [16]), IGFBP-3 (for review [17]) and IGFBP-6 [18], [19] were described to have intracellular functions, the rise in intracellular calcium concentration induced by IGFBPs seems unlikely to be dependent upon their intracellular localization. It is therefore conceivable that such rapid responses occur after the IGFBP cell surface binding onto membrane receptors. Our results strongly strengthen this hypothesis since we demonstrated by two different, but concordant, experimental approaches that most of IGFBPs associate with the surface of MCF-7 and C2 cells suggesting the existence of specific receptors that may account for IGFBP-induced calcium responses. In addition, the fact that IGFBPs dose-dependently increased intracellular calcium concentration also reinforces this hypothesis. Many data support or demonstrate the existence of IGFBP receptors. For example, IGFBP-2 binds to the cell surface through its RGD recognition sequence [20] and its intrinsic mitogenic actions were blocked by a short RGD-containing disintegrin peptide or by a β_1_-integrin receptor blocking antibody [21]. IGFBP-3 was found to have several cell surface binding partners as type V TGF-β receptor [22], [23] or the low-density lipoprotein receptor-related protein-1 (LRP-1)/activated α_2_M receptor [24] (for review [11], [17]). IGFBP-4 was recently shown to interact with a Wnt receptor, Frizzled 8 (Frz8), and a Wnt co-receptor, LRP-6 [25]. Finally, IGFBP-5 would bind onto a serine kinase receptor [26] whereas membrane receptors for IGFBP-6 were not already identified. Nevertheless, none of these membrane receptors have been classically described to induce a transient calcium response. It is therefore conceivable that other IGFBPs’ receptors may exist and be coupled to signaling pathways that trigger intracellular calcium increase. Such hypothesis and characterization is currently under investigation.

Based on our study performed in two different cell models, IGFBP-1 seems to be particular since, contrary to the other five IGFBPs, it never affects intracellular calcium concentration while we showed that it specifically associates with the cell surface. However, IGFBP-1 contains an integrin recognition sequence, Arg-Gly-Asp (RGD), that allows it to bind to the integrin α_5_β_1_ [27] which signaling pathway is implicated in either adhesion, migration, differentiation or apoptosis depending on the cellular context (for review [28]). Even if we cannot generalize to all cell types, one may suggest that the IGFBP-1 functions, mediated through its interaction with integrin α_5_β_1_, such as modulation of cell migration [29], [30], would not require calcium increase although it was described that the activity of FAK, a downstream target of this pair of integrin implicated in cell motility (for review [31]) was modulated by a sustained rise in intracellular calcium concentration [32].

A rapid rise in intracellular calcium concentration induced by an extracellular ligand is often dependent upon a GTPase protein-coupled receptor, GPCR. Therefore, it is conceivable that IGFBPs may associate with specific isoforms of this large receptor family. Interestingly, we and others showed that IGFBP-3 associates with cell surface proteins with an apparent molecular mass of 30–40 kDa, which agrees with that of most GPCR [33], [34]. Totally consistent with this hypothesis, we showed that some IGFBP-induced calcium responses (i.e. IGFBP-2 and -3 in MCF-7 cells, and IGFBP-5 in C2 cells) were sensitive to pertussis toxin, a specific inhibitor of Gi proteins. Moreover, IGFBP-5 stimulates growth of human intestinal muscle cells by activation of Gαi3 [35]. Taken together, these results strongly suggest that GPCR and their associated heterotrimeric GTPase may mediate, at least in part, some IGFBP intracellular actions such as increase of calcium concentration.

In the aim of determining the identity of the IGFBPs’ receptors responsible for such calcium response, one may ask whether there is a common receptor for all IGFBPs or rather a specific receptor for each of them. Despite that IGFBPs possess some conserved amino-acid domains [36]–[39] and were all shown to associate with the surface of MCF-7 cells, the first hypothesis seems unlikely. In fact, in C2 cells, IGFBP-2 and -4 were never detected as being associated with the cell surface. This result would imply that, at a minimum, it would exist, on one hand, a common receptor for IGFBP-1, -3, -5 and -6 and, on the other hand, another one for IGFBP-2 and -4. Moreover, despite their ability to bind to the cell surface, IGFBPs-induced calcium responses were specific, i.e. sensitive or not to pertussis toxin or even totally absent (IGFBP-1 and -5 in MCF-7 cells and IGFBP-1 in C2 cells). Therefore, taken all together, our results strongly indicate that a specific IGFBP receptor may exist for each isoform. Although previously determined for IGFBP-3 [12], the quantification of the IGFBPs’ binding sites and their relative expression levels in each cell type were difficult to address. In fact, the use of specific, and consequently different, primary antibodies does not allow us to quantitatively compare the relative fluorescence intensities measured in each case. Moreover, although cell surface binding was observed by two different technical approaches, they did not permit us to definitively conclude whether this binding is saturable. Thus, experiments conducted with radiolabeled IGFBPs would give more precise results about the association constants and the number of binding sites. Nevertheless, our data provide qualitative results that clearly demonstrate the ability of several IGFBPs to specifically bind to the cell surface of two cellular models.

A particular interest in comparing the IGFBPs cellular responses in two different cellular models lies in the identification of cell specific IGFBPs’ responses (results summarized in Table 1). In fact, for example, IGFBP-5 induces a calcium concentration rise in C2 cells but not in MCF-7 cells. Furthermore, the IGFBP-3-induced calcium response is either sensitive or insensitive to pertussis toxin in MCF-7 and C2 cells, respectively. Such specificity could be the consequence, as mentioned above, of the cell specific expression of particular IGFBP receptors but rather of cell specific couplings between these receptors and cell specifically expressed intracellular G proteins which put in evidence the complexity of these IGFBPs-induced signaling networks. Such cell specific couplings were already described for several GPCRs. In fact, depending of the cell context, glucagon receptor can be either coupled to Gα_s_ or G_q_. Moreover, GLP-1R couples to multiple G proteins, including Gα_s_, Gα_q*/*11_ and Gα_i1,2_ (for review [40]). Consistent with this, it is interesting to note that, on the one hand, the only G protein which has been clearly identified as playing an important role in mediating the effects of an IGFBP, i.e. IGFBP-5, is a Gi protein identified in muscle cells [35] and that, on the other hand, IGFBP-5 increases intracellular calcium concentration in C2 cells, but not in MCF-7 cells, through a pertussis toxin sensitive signaling pathway as shown in the present study. Taken together, and bearing in mind that IGFBP-5 plays crucial roles in muscle cells (for review [41]), these results may suggest that IGFBP-5 acts in the muscular lineage through the specific activation of Gαi3 and the subsequent rise in calcium concentration. When considering the other IGFBPs, the physiological significance of such intracellular calcium increases is somewhat more difficult to determine. Since calcium increase is found in numerous processes, it would account for their described multiple IGF-independent actions such as induction of proliferation, differentiation or modulation of cell metabolism (for review [42]).

**Table 1:** IGFBPs increase intracellular calcium concentration via pertussis toxin-sensitive and -insensitive signaling pathways in MCF-7 and C2 proliferative cells.

IGFBP	MCF-7 cells		C2 proliferative cells	
	Increase of calcium concentration	pertussis toxin	Increase of calcium concentration	pertussis toxin
**IGFBP-1**	−		−	
**IGFBP-2**	+	S	−	
**IGFBP-3**	+	S	+	NS
**IGFBP-4**	+	NS	−	
**IGFBP-5**	−		+	S
**IGFBP-6**	+	NS	+	NS

S: sensitive, NS: non sensitive.

In conclusion, our results clearly reveal rapid and specific molecular actions of IGFBPs in two different cell lines, providing direct evidence of signaling pathways activated by these binding proteins and opening new perspectives for the characterization of the IGF-independent effects of IGFBPs.
